# Inverse Design of Multi-Port Power Splitter with Arbitrary Ratio Based on Shape Optimization

**DOI:** 10.3390/nano15050393

**Published:** 2025-03-04

**Authors:** Yang Liu, Zhe Kang, Haoda Xu, Guangbiao Zhong, Ruitao Zhang, Chaoying Fu, Ye Tian

**Affiliations:** 1 Department of Basic Courses Teaching, Jilin Business and Technology College, Changchun 130507, China; 2 National Key Laboratory of Microwave Photonics, College of Electronic and Information Engineering, Nanjing University of Aeronautics and Astronautics, Nanjing 210016, China; 3 Department of Electrical Engineering and Computer Science, Ningbo University, Ningbo 315211, China; 4 Huzhou Key Laboratory of Medical and Environmental Applications Technologies, School of Life Sciences, Huzhou University, Huzhou 313000, China

**Keywords:** inverse design, silicon photonics, power splitter

## Abstract

Arbitrary ratio power splitters (APSs) play a crucial role in enhancing the flexibility of photonic integrated circuits (PICs) on the silicon-on-insulator (SOI) platform. However, most existing APSs are designed with two output channels, limiting their functionality. In this study, we present a shape optimization method to develop a multiport arbitrary ratio power splitter (MAPS) that enables arbitrary power distribution across three output channels within a compact footprint of 6 µm × 2.7 µm. To validate this approach, two MAPS designs were demonstrated with power ratios of 1:2:1 and 1:2:4. Across a bandwidth range from 1500 nm to 1600 nm, these designs matched the desired power distribution with excess losses (ELs) below 0.5 dB. Experimental results further confirmed the effectiveness of the splitters, with ELs below 1.3 dB over a bandwidth of 1500–1565 nm.

## 1. Introduction

Photonic integrated circuits (PICs) based on the silicon-on-insulator (SOI) platform have garnered significant attention due to their high integration density and compatibility with Complementary Metal-Oxide-Semiconductor (CMOS) technology [1]. Numerous components have been developed on the SOI platform to support the functionality of on-chip PIC systems. Among these, optical power splitters serve as the fundamental building blocks used for light manipulation and are integral to devices such as optical phased arrays [2], modulators [3], and sensors [4]. Traditional power splitters are typically designed to direct signals to two output channels, and various designs have been proposed for arbitrary ratio power splitters (APSs) to enable flexible power distribution [5]. However, the development of a multiport APS (MAPS) holds even greater potential for enhancing PIC system flexibility and facilitating advanced applications such as signal monitoring [6], conversion [7], and more.

Various approaches have been explored to develop MAPSs, including directional couplers (DCs) [8], branch structure [9], photonic crystals (PhCs) [10], multi-layer structures [11], and multimode interference (MMI) couplers [12]. While these methods have demonstrated high-efficiency devices, many require large footprints or complex manufacturing processes to ensure optimal performance. Additionally, MMI couplers with electro-optic tuning elements provide a tunable solution for achieving flexible splitting ratios (SRs) [13]. However, these devices typically occupy hundreds of square microns to mitigate thermal crosstalk. Moreover, conventional design approaches are heavily reliant on the designer’s expertise and involve extensive optimization, further increasing the complexity of the development process.

Recently, inverse design methods have been increasingly employed to create devices with flexible functionalities within compact footprints [14], significantly advancing the development of high-performance MAPS devices. Many studies utilize the direct binary search (DBS) algorithm applied to pixelated structures to achieve target SRs within ultra-compact device dimensions [15,16,17,18,19]. However, the intrinsic cavity structures in these pixelated designs often result in significant propagation scattering, leading to high excess loss (EL). Moreover, these designs demand high-precision fabrication and careful consideration of robustness against etching errors. In Reference [20], a 1 × 5 MAPS was developed using the Powell algorithm applied to a photonic crystal (PhC) structure, achieving a theoretical EL below 0.23 dB over a 40 nm bandwidth. Despite this, the device’s footprint remains relatively large at 41.5 µm^2^. The inverse design of device shapes using various optimization techniques offers an alternative for achieving efficient MAPSs [21,22,23]. However, these designs have primarily been limited to splitters with equal power distribution, leaving the flexibility of such approaches largely unexplored.

In this paper, we propose and experimentally demonstrate a 1 × 3 MAPS with low loss and compact size on the SOI platform. This device is realized through shape optimization of a tapered structure with fixed dimensions of 6 µm × 2.7 µm utilizing the adjoint method. To validate this approach, two MAPS designs with power ratios of 1:2:1 and 1:2:4 were developed. Theoretical analysis indicates that these designs achieve the desired SRs with ELs below 0.5 dB and SR deviations (SDs) below 10% over a broad bandwidth ranging from 1500 nm to 1600 nm. The devices were fabricated using mature 193 nm deep ultraviolet (DUV) lithography through the Multi-Project Wafer (MPW) process. Experimental results demonstrated ELs below 1.3 dB and SDs below 15.8% over the 1500–1565 nm bandwidth.

## 2. Design and Simulations

**Figure 1:**
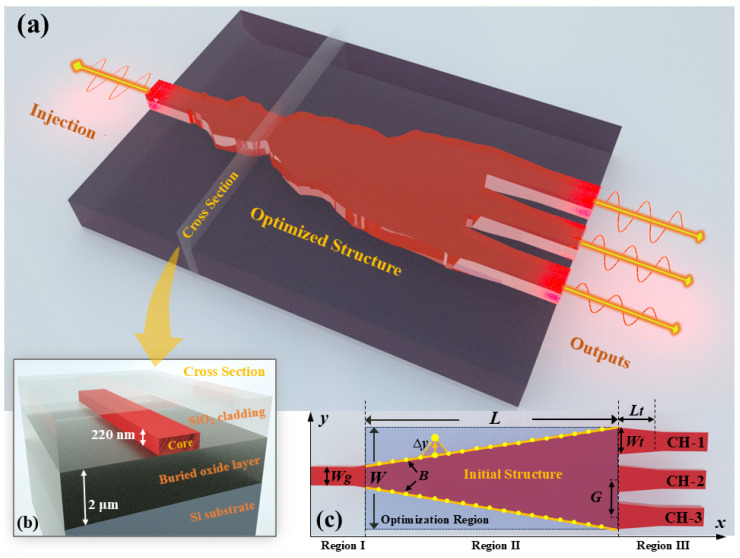
(**a**) A schematic diagram of the proposed MAPS. (**b**) Cross-section of the waveguide. (**c**) The initial structure of the MAPS.

Figure 1a illustrates the three-dimensional diagram of the proposed 1 × 3 MAPS. The device consists of one input waveguide and three output waveguides, each with a width of 0.5 µm ($W_{g}$), and a tapered structure that is 6 µm long (*L*). The boundaries of this tapered structure are optimized through shape optimization. A cross-sectional view of the structure is shown in Figure 1b. The device is designed on an SOI platform, which features a 220 nm thick silicon top layer, a 2 µm buried oxide layer, and a 2 µm silicon dioxide cladding. The initial design of the MAPS is a tapered waveguide with a width linearly varying from 0.5 µm to 2.7 µm (*W*), as shown in Figure 1c. The boundary curve of this structure is parameterized by 200 discrete optimization points (*B*), evenly distributed along the upper and lower boundaries on the x-axis. Additionally, three tapered waveguides with a length of 1 µm ($L_{t}$) and an initial width of 0.7 µm ($W_{t}$) are incorporated between the optimization region and the output waveguides. These additional tapered sections enhance the optical collection area, thereby slightly improving the device’s overall efficiency.

The optimization process aims to achieve the desired SRs for the injected fundamental ${\mathrm{TE}}_{0}$ mode over a broadband range. The figure of merit (FOM) is used to quantify the optimization objective, which is defined as the average transmittance of the output light for each port over the specified bandwidth. The FOM is written as follows:

(1) $$\begin{array}{cc} \mathrm{FOM} & =\sum {\mathrm{FOM}}_{i}\;\;\;\;\;\left(i=1,2,3\right);\\ \mathrm{where}\;\;\;{\mathrm{FOM}}_{i} & =\frac{1}{\lambda_{2}-\lambda_{1}}\int_{\lambda_{1}}^{\lambda_{2}}\left|T_{i,ideal}\left(\lambda \right)\right|d\lambda \\ \; & -\frac{1}{\lambda_{2}-\lambda_{1}}\int_{\lambda_{1}}^{\lambda_{2}}\left|T_{i,ideal}\left(\lambda \right)-T_{i}\left(\lambda \right)\right|d\lambda , \end{array}$$

where $\lambda_{1}$ and $\lambda_{2}$ represent the minimum and maximum wavelengths of the target bandwidth, set at 1500 nm and 1600 nm, respectively. $T_{i,ideal}$ denotes the target transmittance from the specific output channel within this wavelength range. $T_{i}$ is the actual transmittance values measured during the iterations. Notably, a subtraction expression is used in Equation (1). By subtracting the offset from the ideal transmittance, the FOMs directly represent the real transmittance, facilitating real-time monitoring of changes in actual transmittance throughout the iterations.

**Figure 2:**
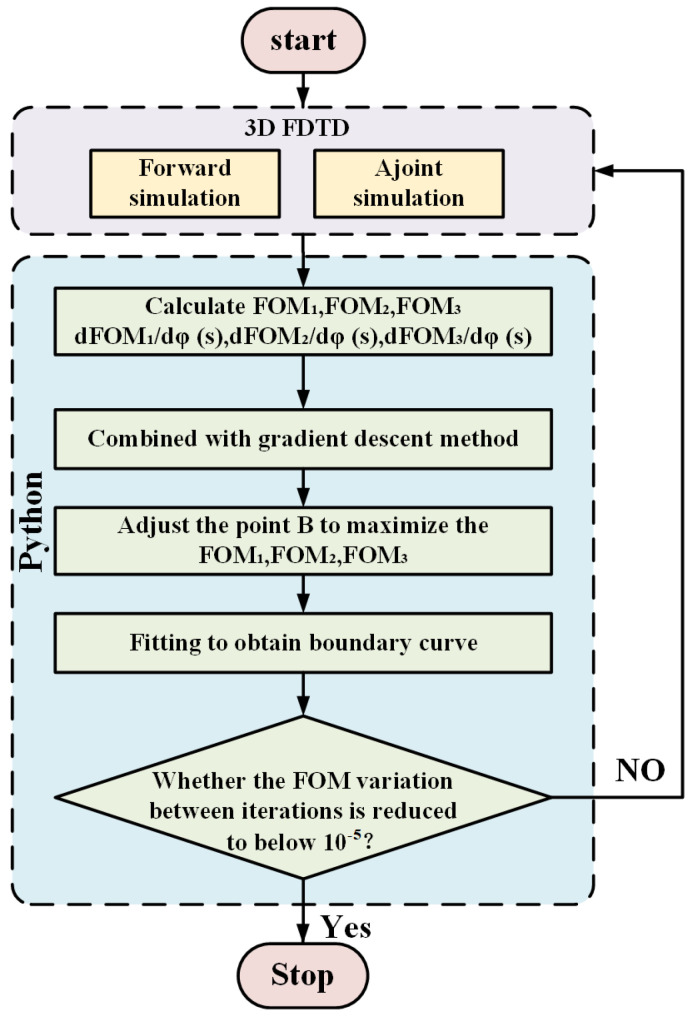
Flow chart of optimizing MAPS using the adjoint method.

To enhance computational efficiency, we employ the adjoint method to optimize the boundary shape. This technique requires only one forward simulation and one adjoint simulation per iteration, providing essential shape gradient information regardless of the number of design parameters [24]. As a result, the number of simulations needed and the overall computational cost are significantly reduced. The optimization process using the adjoint method is depicted in Figure 2. For a certain wavelength, the change of FOM_*i*_ can be expressed as follows [25]:

(2) ΔFOM=2Re∫Δφ(B)ρind(B)·Eadj(B)dA

where Δφ(B) is the deformation in the normal direction at points *B*. dA indicates the differential element along the surface. $\rho^{ind}\left(B\right)$ is the dipole moment caused by the deformation, which can be written as

(3) $$\rho^{ind}\left(B\right)≃\left(\varepsilon_{2}-\varepsilon_{1}\right)\left(E_{∥}\left(B\right)+\frac{D_{⊥}\left(B\right)}{\varepsilon_{2}}\right)$$

where $\varepsilon_{1}$ and $\varepsilon_{2}$ represent the relative permittivity of ${\mathrm{SiO}}_{2}$ and Si, respectively. $E_{∥}\left(B\right)$ and $D_{⊥}\left(B\right)$ represent the tangential component of the electric field and the normal component of the electric displacement at point *B*, which can be obtained by the forward simulation. Meanwhile, the adjoint field $E^{adj}\left(B\right)$ can be split as

(4) $$E^{adj}\left(B\right)=E_{∥}^{adj}\left(B\right)+\frac{D_{⊥}^{adj}\left(B\right)}{\varepsilon_{1}}$$

where $E_{∥}^{adj}\left(B\right)$ and $D_{⊥}^{adj}\left(B\right)$ represent the tangential component of the electric field and the normal component of the electric displacement at *B* given by the adjoint simulation. Thus, the gradient of FOM can be written as follows:

(5) dFOMd(φ(B))=2Re∫[ε2−ε1E∥(B)·E∥adj(B)+1ε1−1ε2D⊥(B)·D⊥adj(B)]dA

The device is modeled using the three-dimensional finite-difference time-domain (3D FDTD) method for both forward and adjoint simulations. At the same time, the gradients of FOMs concerning points *B* are computed using a Python script (V3.6.8). Using the gradient descent method, the y-coordinates of the optimization points are adjusted to maximize the FOM values. To ensure manufacturability, we impose a constraint on the y-coordinate adjustments, limiting the shifting range to ±0.5 µm to prevent the creation of overly sharp or impractical structures. The optimization points are then interpolated using spline curves to generate boundary shapes. This iterative process is repeated until the FOM variation between consecutive iterations falls below 10^−5^, at which point the optimization process is considered converged. When the FOM reaches its maximum, the transmittance $T_{i}$ at each output port converges to the target values $T_{i}^{*}$. By specifying the desired target transmittance values $T_{i}^{*}$ for each port, the optimization points adjust accordingly, resulting in the final structure for the MAPSs.

**Figure 3:**
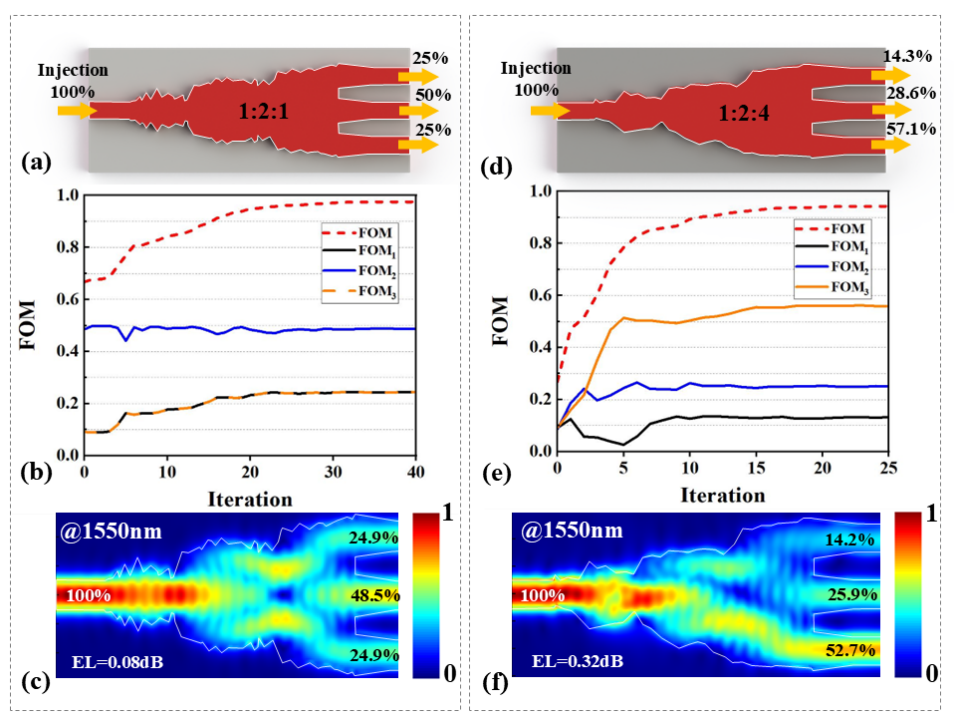
(**a**,**d**) Optimized structures of MAPSs with SRs of 1:2:1 and 1:2:4. (**b**,**e**) FOM evolution during the optimization process for the devices. (**c**,**f**) The simulated optical field distribution for the MAPSs at 1550 nm.

Two MAPS designs with power ratios of 1:2:1 and 1:2:4 were developed to validate this approach, with the optimized structures shown in Figure 3a and Figure 3d, respectively. Here, we define EL and SR deviation (SD) as follows:

(6) $$\begin{array}{cc} \mathrm{EL} & =-10\log_{10}\sum (T_{i}),\\ \mathrm{SD} & =\left|T_{i}-T_{i,ideal}\right|\times 100\%,\;\;\;\;\;\left(i=1,2,3\right) \end{array}$$

where $T_{i,ideal}$ represents the ideal transmittance for each port. The evolution of the FOM for both devices is presented in Figure 3b,e. The FOMs rapidly increase beyond 0.9 within the first 15 iterations, eventually converging to 0.977 for the 1:2:1 MAPS and 0.943 for the 1:2:4 MAPS. It is noteworthy that the optimized FOM for the 1:2:4 MAPS is slightly lower than that of the 1:2:1 MAPS. This discrepancy is attributed to the asymmetry required to achieve a higher extinction ratio between the top and bottom channels. This asymmetry exacerbates boundary ripples, as the length of the optimization region remains unchanged. In future work, this issue could be mitigated by expanding the optimization region for MAPSs.

**Figure 4:**
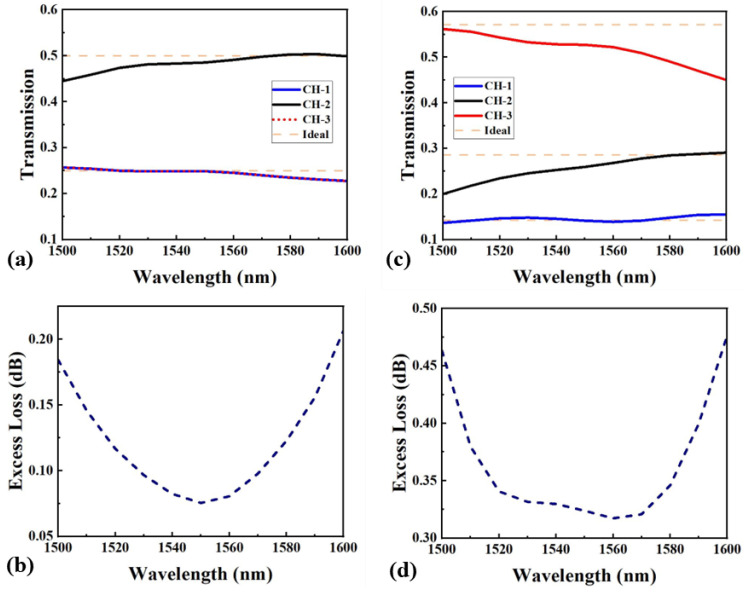
(**a**,**c**) Simulated transmission spectra for 1:2:1 and 1:2:4 MAPSs. (**b**,**d**) The corresponding ELs for 1:2:1 and 1:2:4 MAPSs.

The simulated optical field distributions for the two MAPSs are shown in Figure 3c and Figure 3f, respectively. At 1550 nm, the SR for the 1:2:1 MAPS is 24.9%, 48.5%, and 24.9% (1:1.95:1), with a maximum SD of 1.5% and an EL of 0.08 dB. For the 1:2:4 MAPS, the SR is 14.2%, 25.9%, and 52.7% (1:1.82:3.71), with a maximum SD of 4.4% and an EL of 0.32 dB. The simulated transmission spectra for the two MAPSs over a bandwidth from 1500 nm to 1600 nm are shown in Figure 4a,c. Figure 4b,d depict the corresponding EL for both devices across the entire bandwidth. For the 1:2:1 MAPS, the SD remains within 5.5% for all three channels, and the EL is consistently below 0.2 dB. The 1:2:4 MARS exhibits an SD below 10.1%, with an EL maintained under 0.47 dB across the bandwidth.

**Figure 5:**
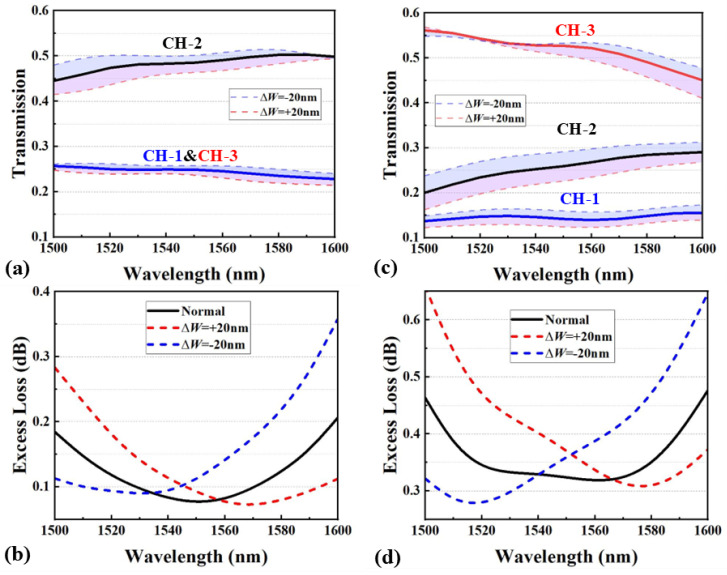
Tolerance analysis: (**a**,**c**) The spectrum variations, and (**b**,**d**) the EL variations for 1:2:1 and 1:2:4 MAPSs with ΔW of ±20 nm.

The fabrication tolerance of the proposed MAPSs was evaluated by varying the device width (ΔW) and analyzing the corresponding changes in the transmission spectrum and ELs across the bandwidth. The width was adjusted by ±20 nm, as 20 nm represents the maximum anticipated mismatch introduced by the 193 nm deep ultraviolet (DUV) lithography process during the multi-project wafer (MPW) run. To modify the device width, either widening or narrowing it, the positions of all optimization points were adjusted simultaneously. Figure 5a,c display the spectrum variations for ΔW ranging from −20 nm to +20 nm. The 1:2:1 MAPS exhibits a variation of less than ±3.8%, while the 1:2:4 MAPS shows a variation of less than ±4%. Figure 5b,d illustrate the effect of ΔW on the ELs. For the 1:2:1 MAPS, the EL spectrum undergoes a blueshift as the device width decreases and a redshift as the width increases, with the EL remaining below 0.36 dB for ΔW variations of ±20 nm. Similarly, for the 1:2:4 MAPS, the EL spectrum follows the same trend, with the EL staying below 0.65 dB. These results indicate that the proposed MAPSs designed by this method possess a certain level of robustness against manufacturing errors.

## 3. Fabrication and Measurement

**Figure 6:**
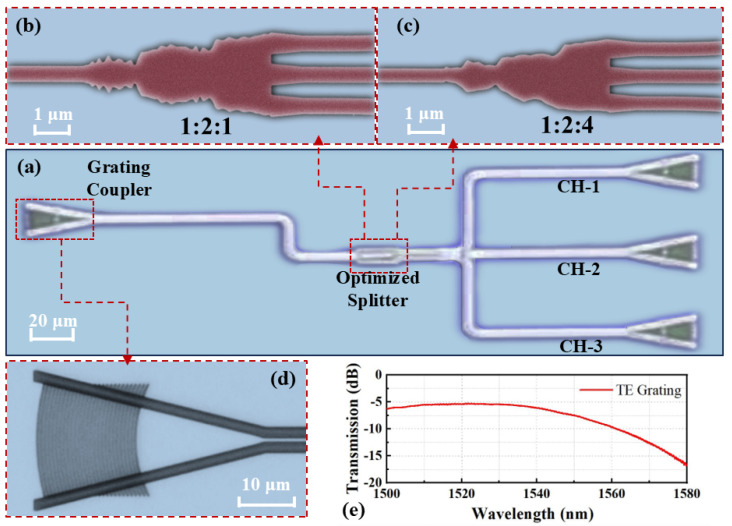
(**a**) On-chip test structure for the MAPSs. (**b**,**c**) The SEM images of the 1:2:1 and 1:2:4 MAPSs. (**d**) Magnified microscope image of the reference GC. (**e**) The measured spectrum of the GC.

The proposed devices were fabricated through a commercial MPW process provided by Zhongke Microelectronics Technology Co. Ltd. (Chengdu, China), using an SOI wafer with a 220 nm top silicon layer and a 2 µm buried oxide layer. Device patterns were defined via 193 nm deep ultraviolet lithography, followed by inductively coupled plasma (ICP) etching to shape the silicon layer. Afterward, a silica upper cladding was deposited using plasma-enhanced chemical vapor deposition (PECVD). Figure 6a presents a microscopic image of the on-chip test structure, while zoomed-in views of the fabricated MAPSs are shown in Figure 6b,c.

In our experimental setup, a tunable laser (Santec TSL-550, Komaki, Japan) served as the light source, with an optical power meter monitoring the transmitted power. Grating couplers (GCs) were employed to couple light into and out of the chip. The GCs were designed with a grating period of 620 nm, an etching depth of 70 nm, and a fill factor of 0.5, as depicted in Figure 6d. The transmission spectrum of the reference GC is provided in Figure 6e, showing a maximum coupling efficiency of −5.27 dB/facet. However, the coupling efficiency declines as the wavelength increases, due to the inherent bandwidth limitations of the GC. Notably, the efficiency drops by approximately 10 dB when the wavelength exceeds 1565 nm, leading to inaccuracies in the measurement. Therefore, our experimental analysis focuses on the narrower wavelength range between 1500 and 1565 nm.

**Figure 7:**
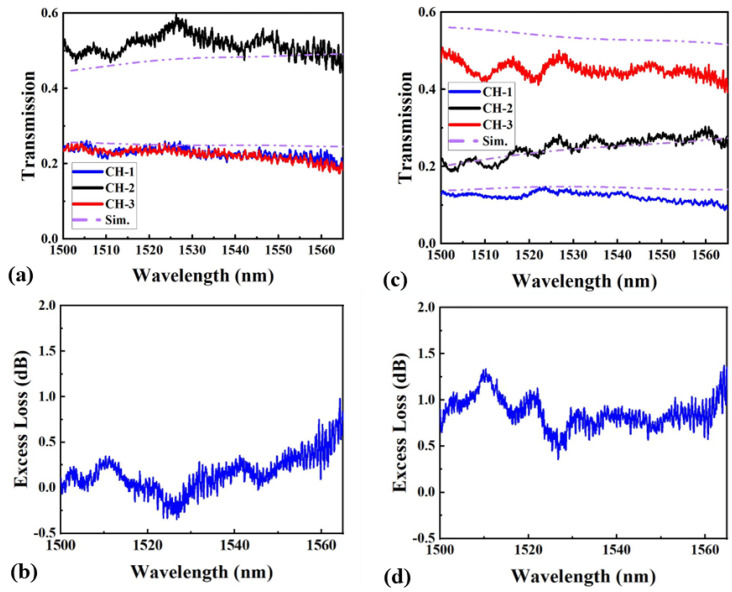
(**a**,**c**) Measured transmission spectra for 1:2:1 and 1:2:4 MAPSs. (**b**,**d**) The corresponding ELs for 1:2:1 and 1:2:4 MAPSs.

The GC losses were deducted from the measured spectra to obtain accurate transmission data. Figure 7a,c displays the normalized transmission spectra for the 1:2:1 and 1:2:4 MAPSs, respectively, over the wavelength range of 1520–1565 nm. We can see that there is generally good agreement between the simulation and experimental results. However, for the 1:2:4 case, a noticeable attenuation in the measured power from CH-3 is observed compared to the numerical analysis. This discrepancy is primarily attributed to manufacturing imperfections caused by the limited precision of DUV lithography, particularly in fabricating sharp ripples along the device boundary. For the 1:2:1 MAPS, the SRs closely match the ideal values, with the SD remaining below 8% for all three channels across the entire bandwidth. The 1:2:4 MARS shows a slightly larger SD of up to 15.8%, primarily due to inconsistencies in the middle channel output. This issue stems from a suboptimal FOM achieved during the optimization process and could be improved by increasing the device dimensions. Figure 7b,d present the corresponding measured ELs for the 1:2:1 and 1:2:4 MAPSs. The 1:2:1 MAPS demonstrates stable performance, with ELs remaining below 0.7 dB across the entire bandwidth. Similarly, the 1:2:4 MAPS maintains ELs below 1.3 dB, indicating that both devices exhibit low losses, though the 1:2:4 MAPS experiences slightly higher losses compared to the 1:2:1 configuration. Despite this, both designs show promising performance, with the potential for further optimization to improve uniformity and reduce loss.

Table 1 presents a comparative analysis of recent MAPS designs developed using inverse design methods on the SOI platform. Notably, most existing research on MAPS focuses primarily on configurations with equal power distribution. In contrast, the design approach presented in this study offers greater flexibility in achieving arbitrary power ratios. Moreover, the proposed method demonstrates superior performance by achieving lower EL and a broader operational bandwidth, while also maintaining a larger minimum feature size (MFS). The larger MFS enhances manufacturability, making the proposed designs more suitable for practical large-scale fabrication.

## 4. Conclusions

In summary, we have demonstrated a novel 1 × 3 MAPS on the SOI platform. By optimizing the device’s shape using the adjoint method, we can achieve arbitrary SRs over a broad bandwidth with low losses. Theoretical validation was performed for MAPS designs with SRs of 1:2:1 and 1:2:4, which were subsequently fabricated using a commercial MPW process. Experimental results revealed that, within the bandwidth of 1500–1565 nm, the EL for all MAPSs remained below 1.3 dB, while the SD was kept below 15.8%. This work demonstrates an efficient approach for designing compact, high-performance MAPSs, paving the way for the development of advanced PIC systems.

## Figures and Tables

**Table 1:** Comparison of inversely designed MAPSs on SOI platform.

Method	Ports	Footprint (µm^2^)	MFS (nm)	SRs	EL (dB)		Bandwidth (nm)	
					**Sim.**	**Exp.**	**Sim.**	**Exp.**
DBS [15]	1 × 3	3.6 × 3.6	120	1:2:1	<1	<1	30	30
DBS [16]	1 × 3	4 × 4	70	1:1:1	<1.9	-	100	-
SO [23]	1 × 3	3.8 × 2.5	100	1:1:1	<0.4	<0.4	150	150
DBS [18]	1 × 3	2.4 × 2.4	90	1:1:1	<0.63	-	30	-
SO [22]	1 × 4	6 × 7.2	150	1:1:1:1	-	<1.08	-	15
PA [20]	1 × 5	41.5 × 41.5	40	1:1:1:1:1	<0.21	-	40	-
				2:3:1:3:2	<0.23	-	40	-
				1:2:1:4:1	<0.18	-	40	-
This Work	1×3	2.7×6	180	1:2:1	<0.2	<0.7	100	65
				1:2:4	<0.47	<1.3	100	65

Sim., simulation; Exp., experimental; SO, shape optimization; PA, Powell algorithm.

## Data Availability

The data presented in this study are available from the corresponding authors upon reasonable request.
